# Mitochondrial morphology in fertile and infertile men: image processing and morphometric analysis of the sperm midpiece

**DOI:** 10.3389/fcell.2025.1609081

**Published:** 2025-06-10

**Authors:** María Fernanda Skowronek, Santiago Pietroroia, Gabriel de Cola, Mauricio Ramos, Diego Silvera, Gabriela Casanova, Federico Lecumberry, Adriana Cassina, Rossana Sapiro

**Affiliations:** ^1^ Unidad Académica de Histología y Embriología, Facultad de Medicina, Universidad de la República, Montevideo, Uruguay; ^2^ Departamento de Procesamiento de Señales, Facultad de Ingeniería, Universidad de la República, Montevideo, Uruguay; ^3^ Unidad de Microscopía Electrónica, Facultad de Ciencias, Universidad de la República, Montevideo, Uruguay; ^4^ Departamento de Bioquímica, Facultad de Medicina, Universidad de la República, Montevideo, Uruguay; ^5^ Centro de Investigaciones Biomédicas, Facultad de Medicina, Universidad de la República, Montevideo, Uruguay

**Keywords:** human infertility, sperm midpiece, sperm mitochondria, fluorescent probes, electron microscopy, image processing

## Abstract

**Introduction:**

The male factor is responsible for 50% of infertility cases. Numerous studies have explored the relationship between human sperm morphology assessed via optical and electron microscopy and reproductive outcomes. In the sperm midpiece, mitochondria are arranged in a helical shape, forming a compact sheath. Disruptions in this precise mitochondrial structure, size, or organization may contribute to infertility. However, despite established links between abnormal sperm morphology and pathology, mitochondrial abnormalities in sperm remain relatively understudied.

**Methods:**

In this study, we employed computational image analysis and fluorescence labelling to quantitatively assess morphometric changes in the sperm midpiece and correlate these findings with mitochondrial ultrastructure in fertile and infertile men.

**Results:**

Our results revealed a significant increase in midpiece area, width, and roundness in sperm from men with teratozoospermia. These findings were further validated by electron microscopy. The ultrastructural morphometric analysis demonstrated disassembled, enlarged, and irregularly shaped mitochondria in sperm from infertile men. Additionally, we applied ultrastructural morphometric analyses to apoptotic sperm samples, observing similar qualitative and quantitative mitochondrial alterations, particularly in those from infertile individuals.

**Discussion:**

Traditional sperm morphology assessments are inherently subjective, but this limitation can be addressed through quantitative morphometric analysis. Enhancing the objectivity and precision of such evaluations is essential for elucidating the biological mechanisms of male infertility and optimizing assisted reproductive technologies. In our study, spermatozoa with poor morphology (<4%) and proximal flagellar abnormalities displayed significantly shorter and wider midpieces. Ultrastructural analysis further revealed that mitochondria in sperm from infertile men were significantly larger and more irregular in shape compared to those from fertile men. These findings indicate an association between altered midpiece morphometry, mitochondrial ultrastructure, and male infertility. The integration of computational tools for automated detection and quantification of these morphological changes offers a promising avenue to improve diagnostic accuracy and deepen our understanding of male reproductive disorders.

## 1 Introduction

Infertility, the failure to become pregnant within a year despite unprotected sexual intercourse, is a widespread problem that affects an estimated 70 million people worldwide (Fainberg and Kashanian, 2019). The World Health Organization (WHO) estimates that the male factor plays a role in 50% of the cases (World Health Organization, 2021).

Standard semen parameters are crucial for the assessment of male fertility and overall reproductive health. They provide important information about the quantity and quality of sperm, but rarely shed light on the causes or indicate possible treatments (World Health Organization, 2021; Björndahl and Kirkman Brown, 2022). Although there is currently controversy (Pelzman and Sandlow, 2024; Keegan et al., 2007), sperm morphology is consistently a good indicator of male fertility. Many authors have even gone so far as to claim that sperm morphology reflects the functional competence of spermatozoa (Coetzee et al., 1998; Menkveld et al., 2011). Even though it should be interpreted in the context of multiple parameters, sperm morphology correlates with sperm fertilization ability in medical-assisted reproduction when strict criteria are applied (Van Waart et al., 2001; Grow et al., 1994; Kruger et al., 1986; Menkveld et al., 1990). Concerning the ultrastructural morphology of the male gamete, sperm functionality appears to be directly related to the integrity of gamete morphology (Bartoov et al., 1999; Reichart et al., 2009). Although attempts have been made to link alterations in subcellular components such as the sperm head (Moretti et al., 2005; Lalonde et al., 1988; Chemes et al., 1999a), acrosome (Lalonde et al., 1988; Albert et al., 1992), and flagellar cytoskeleton (Chemes et al., 1999b; Rawe et al., 2001; Escalier, 2006; Escalier and Serres, 1985) to infertility, the results obtained were not convincing. In previous work, we have described ultrastructural alterations more prevalent in infertile patients than in controls, such as gross alterations of the head and neck and large disorganizations of the axoneme (Skowronek et al., 2010; Skowronek et al., 2012). Despite the many ultrastructural phenotypes responsible for abnormal sperm function and pathology, mitochondrial abnormalities have received relatively little attention (Bartoov et al., 1999; Chemes and Rawe, 2003).

During spermiogenesis, the mitochondria, initially located in the peripheral cytoplasm, migrate and gather around the proximal part of the forming axoneme, which later develops into the sperm midpiece (Lehti and Sironen, 2017). The number of mitochondria varies depending on the species (Gage, 1998; Cummins and Woodall, 1985). They are arranged in a spiral (Ho and Wey, 2007) and form a compact sheath where the mitochondria remain connected by disulfide bridges (Otani et al., 1988; Olson and Winfrey, 1990). Disturbances in this precise arrangement of mitochondria could lead to changes in sperm motility in patients with asthenozoospermia (Mundy et al., 1995). Similarly, the organization, volume, and changes in the ultrastructure of the mitochondrial membrane have also been reported as determinants of flagellar movement and flagellar beating, and therefore associated with asthenozoospermia and infertility (Pelliccione et al., 2011; Cardullo and Baltz, 1991; Folgerø et al., 1993), especially in cases of probable genetic origin (Rawe et al., 2007; Collodel et al., 2011; Moretti et al., 2008).

On the other hand, mitochondria *per se* can undergo morphological changes. Dynamic changes associated with fusion and fission phenomena and apoptosis have been described (Yoon and Wang, 2015). Previously, our group developed a series of scripts to measure morphometric features of the mouse sperm midpiece (SMP) using images obtained by fluorescence microscopy (Skowronek et al., 2025). These tools have proven useful in quantifying the morphometric changes in the mouse sperm midpiece during sperm capacitation. Additionally, we have previously demonstrated that the structure of the human sperm midpiece correlates with semen parameters. Using electron microscopy, we also observed that spermatozoa from samples with a low percentage of normal cells exhibited dilatation of the inner mitochondrial matrix (Irigoyen et al., 2022). In this study, we aim to validate scripts to quantify these variations. We propose that mitochondrial shape alterations can be studied through morphometric analysis of the human sperm midpiece labeled with fluorescent probes. We correlated these results with the quantification of the morphometric features detected by transmission electron microscopy (TEM) in the mitochondria of sperm midpieces from both fertile and infertile men. Our ultimate goal is to identify precise tools to improve the analysis of sperm morphology and explore its implications for male fertility.

## 2 Materials and methods

### 2.1 Subjects and human samples

Men attending the Fertilab andrology clinic for sperm testing and the Unit of Human Reproduction, Pereira-Rossell Hospital Center (Montevideo, Uruguay) for fertility testing were included in the study.

Samples for morphometric analysis by fluorescence microscopy (n = 37) were randomly selected, while samples for TEM analysis (n = 12) were chosen according to the conceiving capacity of each subject.

Inclusion criteria for classifying a man as infertile were: a clinical history with an unknown cause of infertility, an abnormal spermogram, and the absence of a female factor in the couple. None of the infertile men (n = 7) had a child of their own, and all of them had at least two examinations of semen samples showing abnormal sperm morphology according to WHO criteria. Patients with varicocele, history of genital tract infections, endocrine disorders, post-vasectomy control or cryptorchidism were specifically excluded. No genetic or family history of male infertility was identified. Possible causes of infertility in their female partners (ovulatory disorders, anatomical or cervical factors) were also excluded. These men were classified as idiopathic infertility.

Fertile men (n = 5) were selected from age-matched men who had fathered at least one child and presented normal semen parameters.

The semen samples were collected from March 2018 to November 2022 at the School of Medicine, Montevideo, Uruguay.

The Ethics Committee of the School of Medicine, Universidad de la República, Montevideo, Uruguay approved the study. Before sample collection, all participants signed an informed consent form. The laboratory staff assured the anonymity of the participants.

### 2.2 Routine semen analysis

The semen samples were collected after 3 days of sexual abstinence by masturbation in sterile and disposable containers and delivered to the laboratory immediately after ejaculation. The semen was allowed to stand in a laboratory oven (Heratherm™ General Protocol, Thermo Scientific™, Massachusetts, US) at 37°C for 30 min. After liquefaction, the volume, viability, pH, and normal morphology of the semen were analyzed according to WHO guidelines (World Health Organization, 2021). For microscopic analysis, two sperm counting chambers CELL-VU® (Millennium Sciences, Inc., New York, United States) were loaded with each semen sample. Ten different fields per chamber were randomly examined (moving the microscope stage from top to bottom and then to the right, thus avoiding the same optical field) using a Nikon microscope at 37°C. Concentration and motility parameters were analysed using an automated analyzer from SCA-Microoptics (CASA) (Barcelona, Spain) with standard settings according to WHO criteria (World Health Organization, 2021). To assess sperm morphology, semen smears were stained using the Shorr method (IVD: *In Vitro* Diagnostic Medical Device Merck KGaA, Germany) and viewed with a Nikon ECLIPSE E200 bright field microscope at 1000x magnification (under oil immersion). At least 200 consecutive spermatozoa were observed and analyzed per sample. Morphological evaluation was performed in several systematically selected areas of the slide, focusing up and down in different and separated microscopic fields. Following morphological WHO criteria (World Health Organization, 2021) all normal spermatozoa were assessed and scored, and the head, neck-midpiece and tail defects of the abnormal spermatozoa were noted.

Samples were discarded if they showed leukocytospermia (World Health Organization, 2021). The sperm cultures were negative for microorganisms.

### 2.3 Non-apoptotic and apoptotic sperm separation

Semen samples were processed by Magnetic activated cell sorting -MACS. Briefly, first swim-up was performed in PureCeption™ Sperm Washing Medium (SAGE *In Vitro* Fertilization, Inc., California, USA)*.* An aliquot of the swim-up selected sperm was centrifuged, incubated with Annexin V-conjugated microbeads (MiltenyiBiotec, Bergisch Gladbach, Germany), and processed by MACS according to the manufacturer’s (Miltenyi Biotec) and the procedure described by Grunewald and Paasch (2013). The unlabeled cells that ran through the column were collected (non-apoptotic fraction). The magnetic field was removed, and the magnetically retained Annexin V-conjugated sperm cells were eluted (apoptotic selected cell fraction). The sperm chromatin dispersion test was performed in both fractions according to Fernández et al., (2005) *.* An aliquot of each fraction was fixed and processed to be analyzed by TEM.

### 2.4 Evaluation of sperm midpiece morphology by confocal and epifluorescence microscopy

After liquefaction, 37 semen samples were centrifuged at 400 g for 10 min, at room temperature, and the supernatant was discarded. Sperms were resuspended in BWW (Biggers-Whitten-Whittingham) medium (Calvo et al., 1993) and incubated with 50 nM MitoTracker® Red CMXRos - M7512 - (Invitrogen, Waltham, Massachusetts, United States) at 37°C for 30 min in a digital laboratory incubator, Thermo Scientific - Heratherm IMC-18 model. Sperms were then spread on a glass slide and fixed in a mixed solution of 4% w/v paraformaldehyde in 0.1 M phosphate buffer (PB) for 30 min and washed thoroughly in phosphate buffer saline (PBS). Sperm nuclei were counterstained with DAPI (4′,6-diamidino-2′- phenylindole dihydrochloride). After mounting the slides, they were observed using a Leica spectral confocal microscope model TCS SP5 II and a Nikon Eclipse E400 epifluorescent microscope with a 100X, 1.4 NA oil objective (excitation: λ = 488 nm and λ = 543 nm). Several digital images of the spermatozoa of each individual were taken and processed.

### 2.5 Ultrastructural evaluation of sperm mitochondrial morphology by transmission electron microscopy

Semen samples from five fertile control men and seven infertile patients were processed for TEM. Following liquefaction, each sample was centrifuged at 400 g for 10 min. The supernatant was discarded, and the resulting sperm pellet was fixed in 4% (w/v) paraformaldehyde in 0.1 M phosphate buffer (PB, pH 7.4) containing 2.5% (v/v) glutaraldehyde, and stored overnight at 4 °C. Samples were then rinsed in PB (pH 7.4), post-fixed in 1% (w/v) osmium tetroxide for 1 hour, dehydrated through a graded ethanol series and acetone, and embedded in Araldite resin. Polymerization was carried out at 58°C–60°C for 48 h. Sectioning was performed using an RMC MT-X ultramicrotome equipped with a DIATOME diamond knife. Semithin sections (500 nm) were stained with 1% (w/v) toluidine blue and examined under a Nikon ECLIPSE E200 light microscope. Adjacent ultrathin sections (50–70 nm) were stained with uranyl acetate followed by lead citrate and examined using a JEOL JEM-1010 transmission electron microscope operated at 80 kV. For each sample, digital images were captured from at least 100 midpiece-level sections, including both longitudinal (through the central axis of the axoneme) and transverse views. Images were acquired and processed using a Hamamatsu C-4742-95 digital camera to obtain both qualitative and quantitative data on mitochondrial morphology.

### 2.6 Image processing

To analyze the fluorescence and electron microscopy images, we developed two software applications that allowed the analysis of the morphometric characteristics of the sperm midpiece and the mitochondria in an automated and semi-automated manner. For each individual, at least 100 fluorescence images of sperm midpieces and 100 electron microscopy images were quantified. To assess the mitochondrial ultrastructure, only longitudinal sections at the level of the sperm midpiece, passing through the central axis of the axoneme, were analysed. Images were processed using the ImageJ/Fiji (Schindelin et al., 2012) routines implemented in Python (Van Rossum and Drake, 2009).

#### 2.6.1 Fluorescence microscopy image processing

Fluorescence microscopy images were processed as 8-bit images. Noise reduction was obtained by a Gaussian filter, and automatic segmentation of spermatozoa midpieces was obtained from the images labeled with MitoTracker™ using the MaxEntropy thresholding method (Kapur et al., 1985). The MaxEntropy method automatically defines a threshold value *h* in the intensity value that maximises $\psi \left(h\right)$ based on Shannon’s Entropy (Shannon, 1948) where

$$\psi \left(s\right)=\ln\;P_{s}\left(1-P_{s}\right)+\frac{H_{s}}{P_{s}}+\frac{H_{n}-H_{s}}{{1-P}_{s}},\text{with }H_{k}=\sum_{i=1}^{k}p_{i}\ln\;\left(p_{i}\right),P_{s}=\sum_{i=1}^{s}p_{i}$$

$p_{i}=\frac{f_{i}}{N}\;\forall i\in \left\{1,\cdots ,n\right\}$ is the fraction of pixels with value $f_{i}$ , N is the total number of pixels, and n is the total number of grey values observed in the image. Using this method, we maximized the information extracted from the image by enhancing the contrast between the foreground (object) and background distributions. This enabled the automated generation of regions of interest (ROIs) (see Figure 2), from which multiple shape and fluorescence intensity descriptors were calculated. Using a dialog box in ImageJ/Fiji: “Set Measurements” we could select between a lot of morphometric descriptors (area, mean gray value, centroid, X and Y coordinates, center of mass, perimeter, bounding rectangle, fit ellipse, circularity, aspect ratio, roundness, solidity, Feret’s diameter, integrated density, etc, (Schindelin et al., 2012). We used only the parameters that best fit the description of the sperm midpiece and mitochondria. These parameters included morphological features such as area, X and Y coordinates to calculate length and width, roundness, circularity, and solidity, as well as fluorescence metrics such as integrated density and mean intensity to evaluate the fluorescence intensity. All ROIs were user-validated to ensure the selection of isolated and well-defined sperm midpieces. Image processing and segmentation were further supported by additional libraries, including: scikit-image (van der Walt et al., 2014), OpenCV (Bradski, 2000), and SimpleITK (Beare et al., 2018).

#### 2.6.2 Electron microscopy image processing

Electron microscopy images were manually processed, and all mitochondria were individually segmented. Each closed region of interest (ROI) was added to the ROI Manager in ImageJ/Fiji (see Figure 5). These ROIs enabled the automated computation of quantitative morphological parameters (see Supplementary Figure S1). Among the available measurements in ImageJ/Fiji, we selected the following for analysis: *Area*, *Perimeter*, *Circularity*, *Roundness*, *X coordinate,* and *Mean Gray Value*. In ImageJ/Fiji, *Circularity* and *Roundness* (Beare et al., 2018) are calculated as follows:

$$Circularity=\frac{4\;\pi \;Area}{{Perimeter}^{2}},\;Roundness=\frac{4\;Area}{\pi \;M^{2}}$$

where *M* is the major axis of an ellipse fitted to the ROI. Although both parameters sound alike, they have differences. Consider an ellipse and a rectangle ROIs with the same fitted ellipse, they have similar areas, major and minor axes, and angles. Since they have the same major axis (*M*) the *Roundness* is the same for both ROIs, however, the *Circularity* is higher for the ellipse than for the rectangle.

### 2.7 Statistical analysis

Statistical analysis was performed using the GraphPad Prism statistical package version 8.0.1 for Windows, GraphPad Software, San Diego, California, United States, www.graphpad.com. Data were expressed by arithmetic means and the corresponding standard deviation. The normal distribution of the data was tested using the Shapiro-Wilk normality test. Regression lineal analysis was performed between morphometric data and sperm morphology. Comparisons between means were performed using Student’s t-test or Mann-Whitney test, depending on the normal distribution of the data (two groups). Hypotheses were compared with two tails, and a p-value of less than 0.05 was considered statistically significant.

## 3 Results

### 3.1 Clinical data and semen analysis

A total of 49 men aged 21–50 years were included in the study. Of these, 25 were normozoospermic, and 24 had abnormal semen parameters, including asthenozoospermia (n = 2), teratozoospermia (n = 4), oligozoospermia (n = 6), oligo-asthenozoospermia (n = 1), terato-asthenozoospermia (n = 2), oligo-terato-asthenozoospermia (n = 5), and oligo-teratozoospermia (n = 4).

The age of the participants and semen characteristics analyzed in the morphometric study using fluorescence microscopy are presented in Table 1 and Supplementary Data S1 n = 37. Age and semen characteristics from five fertile and seven infertile men analyzed by TEM are shown in Table 2. Descriptive statistics (mean, standard deviation, median, minimum, and maximum) for sperm concentration, progressive motility, and normal morphology are reported for participants in both experiments (Tables 1, 2).

**TABLE 1:** Age and descriptive characteristics of subjects’ sperm parameter**s**.

Parameter	Mean	SD	Median	Max	Min	Lower reference value (WHO, 2021)
Subject age (yr)	35.4	5.8	36.0	50.0	21.0	—
Sperm concentration (million/mL)	45.5	38.4	35.0	159.0	1.6	16.0
Progressive Motility (a + b) (%)	47.5	20.3	52.0	82.0	3.0	30.0
Normal Sperm Morphology (%)	6.6	3.4	7.0	15.0	0.0	4.0
Alterations of the sperm head (%)	34.9	11.4	35.0	60.0	16.0	—
Alterations of the sperm midpiece (%)	1.9	1.4	2.0	5.0	0.0	—
Alterations of the sperm principal piece (%)	2.7	2.1	3.0	8.0	0.0	—

Data were obtained by analyzing the first spermogram of 37 individuals who attended the andrology clinic. SD, standard deviation; Max, maximum value; Min, minimum value.

**TABLE 2:** Age and descriptive characteristics of semen parameters belonging to fertile and infertile men analyzed by TEM.

Parameter	Fertile men					Infertile men					
	Mean	SD	Median	Max	Min	Mean	SD	Median	Max	Min	Lower reference value (WHO, 2021)
Men age (yr)	42.2	2.3	43.0	44.0	38.0	34.7	4.8	35.0	44.0	29.0	—
Sperm concentration (million/mL)	76.2	56.1	71.0	147.0	12.0	71.2	97.1	21.3	214.0	6.9	16.0
Progressive Motility (a+b) (%)	58.0	14.6	63.0	71.0	35.0	50.3	23.8	54.0	74.0	13.0	30.0
Normal sperm morphology (%)	20.4	5.6	21.0	28.0	14.0	2.9	1.9	2.0	7.0	1.0	4.0

Data were obtained by analyzing the first spermogram of 12 individuals who attended the andrology clinic. SD, standard deviation; Max, maximum value; Min, minimum value; LRV, Low reference Value (World Health Organization, 2021).

### 3.2 Analysis of the sperm midpiece by confocal or epifluorescence microscopy

**FIGURE 1:**
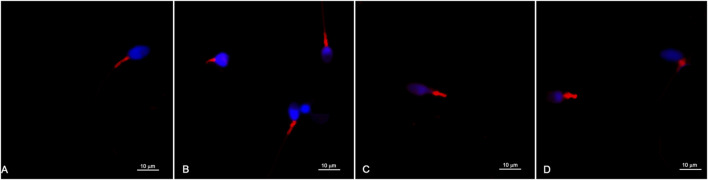
Representative images of sperm labelled with MitoTracker™ and observed with a confocal microscope **(A**–**D)**. The red fluorescent labelling is confined to the sperm midpiece and different marking patterns are observed. The sperm head and nuclear chromatin are labelled in blue.

Great heterogeneity was observed in the morphological characteristics and dimensions of the sperm midpieces. Most sperm midpieces were labelled with MitoTracker™, and the red fluorescent labelling was specifically confined to this sperm region (Figure 1).

Different labeling patterns were observed, indicating a large heterogeneity in the morphometry of the midpiece of human spermatozoa and possible differences in mitochondrial organization. Details in the distribution of red fluorescence within the sperm midpiece can also be observed (Figures 1A–D).

### 3.3 Morphometric analysis of the sperm midpiece

**FIGURE 2:**
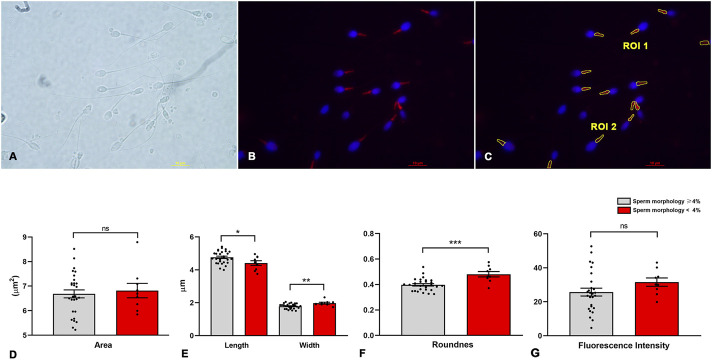
Image processing and morphometric analysis of the sperm midpiece according to sperm morphology. **(A)** Bright field. **(B)** Sperm midpieces labelled in red with MitoTracker™, the nucleus is shown in blue. **(C)** Segmentation of the sperm midpiece and determination of Region of Interest (ROI). **(D–G)** Morphometric parameters of the midpiece compared to sperm morphology (< or ≥4%) (n = 9 and n = 28 respectively) according to WHO criteria. t-test - *p < 0.05.

To determine the morphometric characteristics of the sperm midpieces, we developed a script that automatically detects the MitoTracker™-labeled areas, segments them, and generates ROIs for analysis (Figure 2 and Supplementary Data S1).

Initially, we manually verified that MitoTracker™ specifically labeled the midpiece, allowing accurate identification of the mitochondrial region along the flagellum (Figures 2A–C). Next, we assessed the relationship between conventional sperm morphology (strict criteria) and morphometric measures obtained from our script (Figure 2 and Supplementary Data S1). Linear regression revealed no significant association between sperm morphology and either the mean area (R^2^ = 0.013, p = 0.5) or the mean fluorescence intensity (R^2^ = 0.025, p = 0.4) of ROIs in samples from the 37 men. However, negative correlations were found between the percentage of morphologically normal sperm and midpiece roundness (R^2^ = 0.37, *p* = 0.0001), length (R^2^ = 0.14, *p* = 0.03), and width (R^2^ = 0.27, *p* = 0.001). Samples were further grouped based on global sperm morphology, using WHO strict criteria and a 4% threshold. We included 28 samples with ≥4% normal morphology and 9 samples with <4%. No difference in midpiece area was observed between groups (6.68 ± 0.86 vs. 6.82 ± 0.88 µm^2^; *p* = 0.68; Figure 2D). However, samples with <4% normal morphology had wider and rounder midpieces (width: 1.97 ± 0.16 vs. 1.79 ± 0.15 µm, *p* = 0.0042; roundness: 0.48 ± 0.06 vs. 0.40 ± 0.05, *p* = 0.0002), along with shorter lengths (4.4 ± 0.42 vs. 4.8 ± 0.38 µm, *p* = 0.026; Figures 2E,F). No differences were found in circularity, solidity, or fluorescence intensity (31.6 ± 7.3 vs. 25.7 ± 12.2; *p* = 0.18; Figure 2G).

**FIGURE 3:**
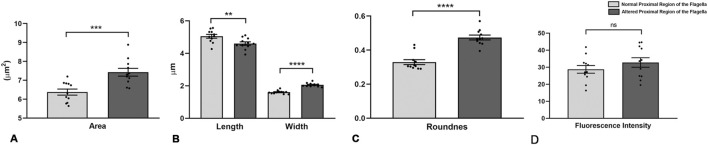
Morphometric analysis of human sperm midpieces according to the morphology of the proximal region of the sperm flagella. **(A–D)** Morphometric descriptors of the midpiece according to normal or altered morphology of the proximal region of the sperm flagella. n = 11 (7 normozoospermic samples and 4 teratozoospermic samples, according to WHO criteria). t-test - *p < 0.05.

To further investigate midpiece morphology, we manually classified sperm from eleven subjects (7 normozoospermic and four teratozoospermic) as having either a normal (NPF) or altered (APF) proximal flagellar region (Figure 3 and Supplementary Data S2). For each subject, 50–100 sperm images were analyzed. Sperm with APF exhibited increased midpiece area (7.42 ± 0.69 vs. 6.37 ± 0.53 µm^2^, *p* = 0.0007), width (2.06 ± 0.12 vs. 1.61 ± 0.11 µm, *p* < 0.0001), and roundness (0.47 ± 0.05 vs. 0.33 ± 0.005, *p* < 0.0001), as well as decreased length (4.60 ± 0.35 vs. 5.05 ± 0.39 µm, *p* = 0.0097; Figures 3A–C). No differences were observed in circularity, solidity, or mean fluorescence intensity (32.8 ± 9.3 vs. 28.7 ± 7.5; *p* = 0.28; Figure 3D).

### 3.4 Analysis of the sperm midpiece by transmission electron microscopy

**FIGURE 4:**
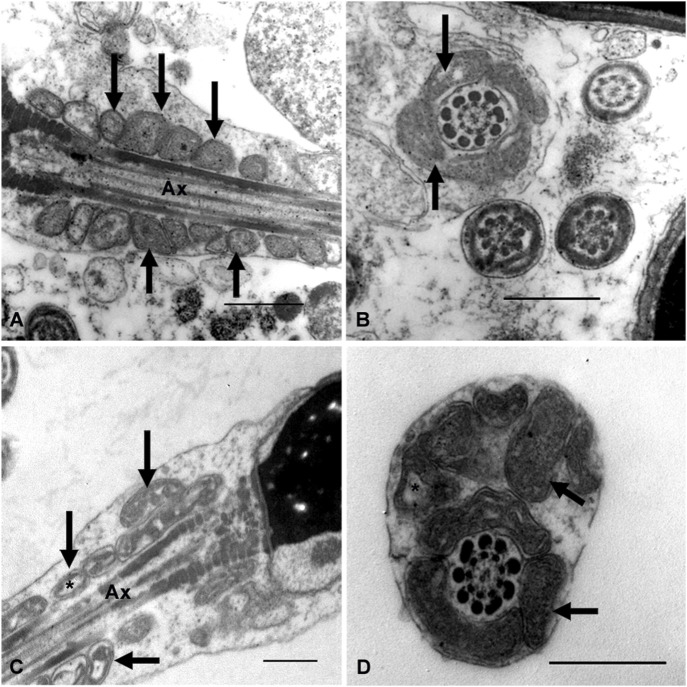
Ultrastructural characteristics of the sperm midpiece from fertile and infertile men. Different patterns of mitochondrial organisation and morphology are observed (arrows). Mitochondria in **(A,B)** are smaller, with more homogeneous profiles and regularly arranged around the axoneme. **(C,D)** show mitochondrial disorganisation about the axoneme, dilated and irregularly shaped mitochondria. Clear spaces between the mitochondrial membranes (*) could suggest edema or mitochondrial swelling. Ax = axoneme. Bars = 500 nm.

The ultrastructural analysis was performed on samples from seven subjects with infertility and five fertile men. Images of at least 100 sections were analyzed at the level of the sperm midpiece, both longitudinally and transversely. In some of the analyzed sections, the mitochondria were round, regular, and well-organized around the axoneme. Their cristae are easily recognizable, and the regular separation of the outer and inner mitochondrial membranes can be observed (Figures 4A, B). In other sections, these organelles show abnormal profiles with irregular shapes, a disorganized arrangement around the axoneme, and large electron-lucent areas in the mitochondrial matrix that often prevent the cristae from being visible (Figures 4C, D). Although normal (Figures 4A, B) and altered mitochondrial profiles (Figures 4C, D) were observed in fertile and infertile men, the latter group had the highest proportion of abnormal forms.

### 3.5 Ultrastructural morphometric analysis

**FIGURE 5:**
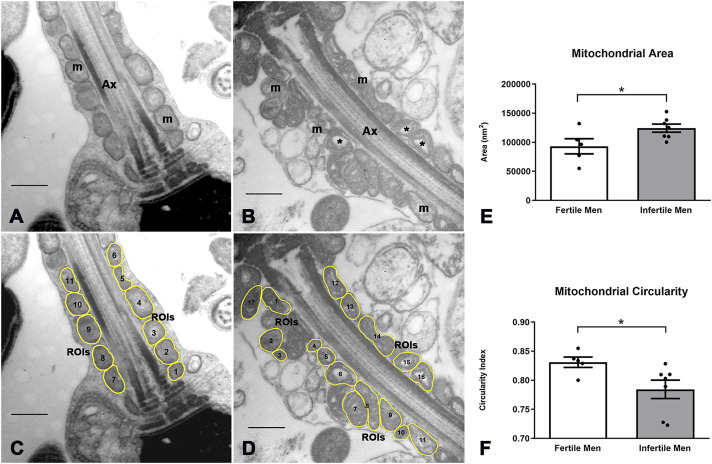
Image processing and morphometric analysis of the mitochondrion in the sperm midpiece of fertile and infertile men. **(A,B)** Ultrastructural characteristics of the mitochondria in fertile and infertile men, respectively. **(C,D)** Segmentation of the mitochondrion in the sperm midpiece and determination of ROIs. **(E,F)** Quantitative analysis of morphometric descriptors. n = 5 fertile men and n = 7 infertile men. t-test - *p < 0.05. Bars 500 nm m = mitochondria, Ax = axoneme, * = mitochondrial matrix dilation.

To perform the ultrastructural morphometric analysis, we developed a new script that semi-automatically recognizes the mitochondria in the sperm midpiece and generates image descriptors. Segmentation was performed manually, and ROIs were defined (Figures 5A–D). The descriptors for shape and size were determined by the script (Figures 5E, F and Supplementary Data S3). To perform the morphometric study, only the mitochondria in the longitudinal sections passing through the central axis of the axoneme were analyzed (almost 100 sections and a total of about 1,000 mitochondria per subject).

A quantification analysis of the observed characteristics confirmed an increase in mitochondrial size and heterogeneity in infertile men. The mean ± SD of mitochondrial area was smaller in fertile than in infertile men (93.085 ± 28.907 vs. 124.191 ± 18.471 nm^2^, p = 0.045) (Figure 5E), while the mean ± SD of mitochondrial circularity was higher in fertile men (0.83 ± 0.02 vs. 0.78 ± 0.04, p = 0.046) (Figure 5F). No differences were observed for other mitochondrial morphological parameters (perimeter, mean gray value, X coordinate).

### 3.6 Mitochondrial morphology in apoptotic spermatozoa

**FIGURE 6:**
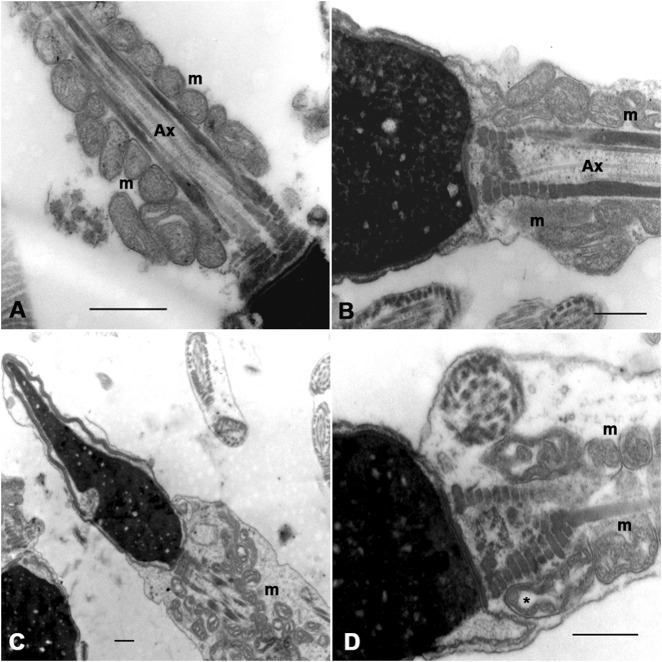
Sperm mitochondrial morphology in spermatozoa subjected to MACS (Magnetic Cell Sorting). In non-apoptotic sperm **(A,B)** the mitochondria (m) are arranged around the axoneme (Ax) and show no gross changes in the organization of the inner membranes. In apoptotic sperm **(C,D)**, dispersed and swollen mitochondria (m) are observed, heterogeneous in shape and size, with gross dilations between the mitochondrial membranes (*). Bars: 500 nm.

We proposed to analyze morphological changes in the mitochondria in spermatozoa subjected to a sperm selection method (magnetic activated cell sorting -MACS) that can separate apoptotic and non-apoptotic spermatozoa. We use the same scripts and computer programs developed by our group to analyze and quantify these morphological changes. Semen from five infertile men with a total sperm count of more than 20 million sperm (an amount high enough to perform TEM), normal sperm morphology between 1% and 7% and sperm DNA fragmentation, measured by the sperm chromatin dispersion (SCD) assay, of more than 16%, underwent the separation method based on the use of annexin V columns. The ultrastructural analysis of these apoptotic and non-apoptotic spermatozoa is shown in Figure 6. Changes similar to those described above were observed. In non-apoptotic spermatozoa, mitochondria were mainly arranged around the axoneme and were homogeneous in shape and size (Figures 6A, B). In apoptotic spermatozoa, mitochondria appeared mostly disorganized around the axoneme, larger and more heterogeneous than in non-apoptotic spermatozoa (Figures 6C, D). Gross dilations, or clear spaces between mitochondrial membranes, were observed more frequently (Figure 6D).

**FIGURE 7:**
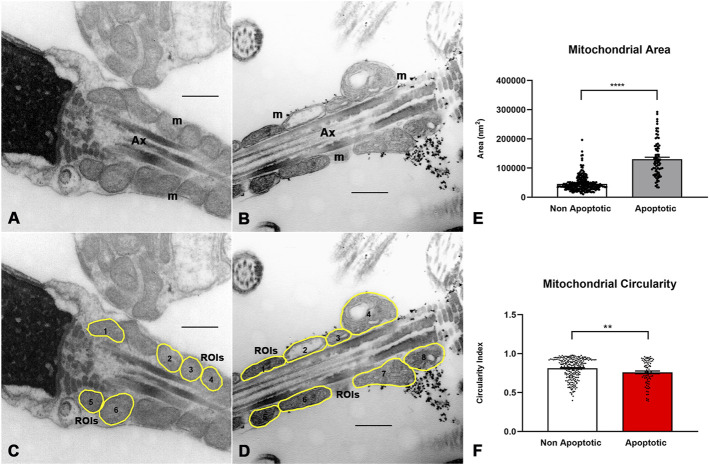
Image processing and morphometric analysis of the mitochondrion in the sperm midpiece of non-apoptotic and apoptotic spermatozoa. **(A,B)** Ultrastructural features of the mitochondria in non-apoptotic and apoptotic spermatozoa respectively. **(C,D)** Segmentation of the mitochondrion in the sperm midpiece and determination of ROIs. **(E,F)** Quantitative analysis of morphometric descriptors. Mann Whitney test, n = 251 non apoptotic sperm and 82 apoptotic sperm. p < 0.05. Bars 500 nm m = mitochondria, Ax = axoneme.

The morphometric analysis of the manually determined ROIs is shown in Figure 7 and Supplementary Data S3. The mean ± SD of mitochondrial area was smaller in non-apoptotic than in apoptotic spermatozoa (45.651 ± 25.334 vs. 130.228 ± 62.117 nm^2^, p < 0.0001, n = 251/82) (Figure 7E), whereas the mean ± SD of mitochondrial circularity was higher in non-apoptotic spermatozoa (0.81 ± 0.13 vs. 0.76 ± 0.15, p = 0.005, n = 251/82) (Figure 7F), which is consistent with the previously described ultrastructural differences between fertile and infertile males. No differences were found in other morphometric descriptors (mean gray value).

## 4 Discussion

The evaluation of human sperm morphology provides important information on the functional status of the male reproductive organs, especially the testes and epididymis (Coetzee et al., 1998; van der Merwe et al., 2005; Esteves, 2022). Despite its historical relevance, the clinical utility of sperm morphology analysis is currently being reassessed, with conflicting evidence regarding its predictive value for pregnancy outcomes in patients with teratozoospermia undergoing assisted reproductive technologies (Pelzman and Sandlow, 2024; Keegan et al., 2007; Danis and Samplaski, 2019; Zhou et al., 2021). The morphological analysis of sperm using routine procedures is associated with a certain subjectivity that can be overcome using quantification techniques. Routine analyses often focus on overall sperm shape, but more detailed morphometric approaches can quantify specific subcellular regions. Improving the accuracy and objectivity of morphological analysis can help elucidate the mechanisms underlying infertility and support various assisted reproductive techniques (Khatun et al., 2018).

In this study, we have analyzed the morphology of the midpiece and sperm mitochondria. Although the association between changes in mitochondrial morphology and infertility is well known (Chemes and Rawe, 2003; Pelliccione et al., 2011; Rawe et al., 2007), there is a lack of systematization of the characteristics of the organelle in males. We present a novel approach based on the automated and systematic analysis of fluorescence images, specifically labelling the sperm midpiece and extracting morphometric descriptors. Delineation of the sperm midpiece and determination of its dimensions is difficult with conventional light microscopy and labelling routine methods. The use of a fluorescent marker specifically directed at the mitochondria, such as MitoTracker™, improves the procedure. MitoTracker™ diffuses passively through the plasma membrane and accumulates in active mitochondria. The dye is permanently bound to the mitochondria and remains after the cell has been fixed (Chazotte, 2009; Poot et al., 1996) so that changes in the midpiece accurately reflect modifications in the morphology of the organelles. The specific labelling of the midpiece allows, through the use of ImageJ/Fiji (Schindelin et al., 2012) and a segmentation protocol, the identification of ROIs from which morphometric descriptors were extracted. Using our custom-developed script, we analyzed large sets of images in a fast, automated, accurate, and reproducible manner, generating a rich dataset of quantitative descriptors. In manually segmented sperm midpieces, we previously observed a correlation between the length and width of the midpiece and normal sperm morphology according to WHO criteria (Irigoyen et al., 2022). Consistent with these findings, we now observed significant differences in midpiece dimensions: spermatozoa with poor morphology (<4%) and characteristic malformations of the proximal flagellum exhibited shorter and wider midpieces.

These results support earlier findings reported by other authors, correlating specific morphometric parameters with male fertility status (García-Vázquez et al., 2016; Mossman et al., 2013). For example, higher sperm head width and lower length/width ratios were detected in spermatozoa from fertile than infertile men (Morales et al., 1988; Boyle et al., 1992). Mean flagellum length and the mean total sperm length were positively associated with semen characteristics measured manually (Mossman et al., 2013).

Sperm organelles, such as mitochondria, are difficult to visualize using standard staining techniques or optical microscopy due to their small size and complex structure. Electron microscopy offers a high-resolution alternative that allows for detailed examination of these organelles (Baccetti et al., 1995). Numerous ultrastructural studies have attempted to detect subcellular changes, particularly in the flagellum, which could clarify the mechanisms underlying alterations in sperm parameters and, thus, infertility (Bartoov et al., 1999; Reichart et al., 2009; Chemes et al., 1999b; Chemes and Rawe, 2003; Rawe et al., 2007; Berkovitz et al., 1999; Rawe et al., 2000; Baccetti et al., 2002; Curti et al., 2014). However, pathologies affecting the sperm midpiece and mitochondrial sheath have received less attention (Chemes and Alvarez, 2012). In the present study, we proposed a systematic computational analysis of the morphology of the sperm mitochondria. In contrast to other studies (Mundy et al., 1995; Pelliccione et al., 2011; Baccetti et al., 1995), we used image processing to obtain various morphometric descriptors and measurements of the size (area) and shape (roundness) of each mitochondrion in the sections of the sperm midpiece. As in other studies, we observed swollen and irregularly assembled mitochondria, but our method allowed us to demonstrate quantitatively that mitochondria were significantly larger and more irregularly shaped. These findings align with analysis under the fluorescence microscope, where sperm classified as having poor morphology according to WHO criteria exhibited larger and broader midpieces.

Although automation through CASA has the potential of improving the morphometric analysis of sperm, the evaluation of sperm morphology by CASA is not yet an established method (Esteves, 2022). The integration of machine learning and deep learning offers new opportunities to enhance classification accuracy. These tools are starting to be applied in sperm classification (Marín and Chang, 2021; Chandra et al., 2022; Mahali et al., 2023; Riordon et al., 2019). Given the heterogeneity in sperm morphology—ranging in shape, size, and texture—analysis tools must be adaptable (Shaker et al., 2017). The open-source nature of ImageJ/Fiji allowed us to tailor our segmentation scripts to address these complexities. Our approach minimizes user-dependent variability and converts qualitative assessments into reliable, objective data. These tools facilitated automated and semi-automated extraction of morphometric descriptors in a reproducible and objective manner. The collection and processing of large numbers of images with minimal human intervention strengthened the reliability and credibility of our results. Our algorithm converts qualitative observations of the sperm midpiece and mitochondria into objective numerical data that validates and supports our conclusions.

Apoptosis in the spermatozoon is questioned and the morphological changes in the mitochondria during this process are poorly known. Interestingly, apoptotic spermatozoa displayed large and irregularly shaped mitochondria similar to those observed in infertile men. The presence of spermatozoa with submicroscopic features similar to those of somatic apoptosis has been reported in human ejaculates (Baccetti et al., 1996). Many studies have linked ultrastructural sperm defects to human sperm apoptosis and pathology (Baccetti et al., 1995; Curti et al., 2014; Baccetti et al., 1996). Specifically, the sperm midpiece in apoptotic cells frequently contains swollen and poorly organized mitochondria (Engel et al., 2018; Grunewald et al., 2017; Muratori et al., 2000). In the early stages of apoptosis, phosphatidylserine is externalized to the outer leaflet and can bind to annexin V. Annexin V, coupled with submicroscopic, biodegradable superparamagnetic beads (MACS), has been used to separate apoptotic from non-apoptotic sperm when applied to a column exposed to a strong magnetic field (Said et al., 2005; Esbert et al., 2017; Lin et al., 2019). The method is considered sufficiently specific to separate both types of cells and has already been used to correlate apoptosis to sperm morphology (Aziz et al., 2007). Morphometric analysis of apoptotic spermatozoa, selected using this specific enrichment technique, revealed a similar frequency and pattern of mitochondrial defects to those seen in samples from men with unexplained infertility. Consequently, the algorithm developed here holds promise for application in diverse contexts beyond infertility, including the assessment of sperm quality and apoptosis. The current data highlight the algorithm’s potential as a robust tool for the quantitative analysis of mitochondrial morphology.

While TEM remains a gold standard for ultrastructural assessment, its cost and labor intensity limit its clinical application (Chemes and Rawe, 2003). Our semi-automated method offers a scalable alternative that retains analytical depth. Measuring and analyzing large numbers of mitochondria with minimal user intervention represents a significant advancement in the study of sperm morphology and male infertility. Nevertheless, validation in larger and more diverse cohorts is essential.

A limitation of the study is the lack of a functional analysis to explain the presence of altered sperm mitochondria in the ejaculate, which may trigger mitochondrial malformations. For example, it is known that oxidative stress triggers mitochondrial swelling, reduced mitochondrial membrane potential, release of cytochrome C, activation of caspases, and apoptosis (Teranishi et al., 2000; Karbowski et al., 1999). This mechanism could underlie both the sperm alterations and the infertility in the individuals studied. To investigate this further, future studies should include functional assessments of mitochondrial activity, measurements of oxidative stress markers in seminal fluid, and correlation with morphometric parameters.

Another important limitation of the study is the sample size. Though the number of patients is adequate as a tool to prove the method to achieve mitochondrial and midpiece morphometric analysis, it requires validation in larger cohorts. Specifically, the size of the group of fertile and infertile men in the TEM analysis limits the universality of the conclusions to other types of male infertile conditions other than idiopathic, e.g., genetic disorders or mitochondrial diseases. Forthcoming studies with diverse populations, larger sample sizes, and specific groups of patients are needed to confirm these findings and their diagnostic value.

Although our findings indicate a higher incidence of mitochondrial and midpiece abnormalities in teratozoospermic subjects, the teratozoospermia is primarily attributable to morphological defects in the sperm head (Table 1 and Supplementary Data S1), rather than in the midpiece. We propose that the mitochondrial alterations observed are not a direct consequence of an increased prevalence of midpiece abnormalities. This represents a potential limitation, as the ultrastructural mitochondrial analyses were not specifically conducted on spermatozoa with confirmed midpiece defects. Nevertheless, when we used image analysis to select spermatozoa exhibiting an altered morphology of the flagellum, morphometric alterations in the sperm midpiece became more significant. To clarify this issue, the goal would be to perform morphometric analyses using both fluorescence microscopy and electron microscopy on the same sample. The implementation of single-cell selection or analysis techniques specifically targeting spermatozoa with midpiece abnormalities, along with the adoption of advanced high-resolution microscopy technologies capable of examining cells *in vivo*, would represent important objectives for future research.

## 5 Conclusion

The morphological remodeling of mitochondria reflects detectable changes within the sperm midpiece that appears to be closely associated with impaired sperm quality and male infertility. In this study, we introduced a novel, automated image analysis pipeline capable of quantifying sperm midpiece and mitochondria morphology alterations with high precision and reproducibility. Our results demonstrated that infertile men and apoptotic spermatozoa share similar mitochondrial abnormalities—namely, increased size and irregular morphology—suggesting a potential link between mitochondrial dysfunction, apoptosis, and male infertility.

Our study demonstrates that computational morphometric analysis of sperm midpieces and mitochondria provides valuable, objective data that correlate with sperm quality and fertility status. This methodology could support clinical diagnostics, assist in evaluating sperm apoptosis, be useful for the development of targeted treatments, and potentially serve as a training foundation for AI-based classification systems. Future work combining morphometric, functional, and genomic data will be critical for advancing our understanding of male infertility.

## Data Availability

Scripts and a minimal working example image dataset are publicly available at https://gitlab.fing.edu.uy/imagina/mitomorph/.

## Supplementary material

SUPPLEMENTARY FIGURE S1 Representative block diagram of image processing.

SUPPLEMENTARY DATA S1 Semen parameters including sperm abnormalities, defect type and measures of morphometric ROIs under MO.

SUPPLEMENTARY DATA S2 Total data of morphometric study of 11 samples analyzed under MO according the alteration of flagella.

SUPPLEMENTARY DATA S3 Electron microscopy analysis. Raw data of seminal characteristics of fertile and infertile men (n=12). TEM data of apoptotic and non-apoptotic sperm.

## Abbreviations

WHO, World Health Organization; TEM, Transmission Electron Microscopy; SMP, Sperm Midpiece; PB, Phosphate Buffer; PBS, Phosphate Buffer Saline; BWW, (Biggers-Whitten-Whittingham) Medium; ROI, Region of Interest; SD, Standard Deviation; FL Images, Fluorescent Images; NPF, Normal Proximal region of the Flagella; APF, Altered Proximal region of the Flagella; Ax, Axoneme; SCD, Sperm Chromatin Dispersion.

## References

[B1] Albert M. Gallo J. M. Escalier D. Parseghian N. Jouannet P. Schrevel J. (1992). Unexplained *in-vitro* fertilization failure: implication of acrosomes with a small reacting region, as revealed by a monoclonal antibody. Hum. Reprod. 7 (9), 1249–1256. 10.1093/oxfordjournals.humrep.a137836 1479007

[B2] Aziz N. Said T. Paasch U. Agarwal A. (2007). The relationship between human sperm apoptosis, morphology and the sperm deformity index. Hum. Reprod. 22 (5), 1413–1419. 10.1093/humrep/dem016 17303629

[B3] Baccetti B. Bernieri G. Burrini A. G. Collodel G. Crisà N. Mirolli M. (1995). Notulae seminologicae. 5. Mathematical evaluation of interdependent submicroscopic sperm alterations. J. Androl. 16 (4), 356–371. 10.1002/j.1939-4640.1995.tb00541.x 8537254

[B4] Baccetti B. Capitani S. Collodel G. Strehler E. Piomboni P. (2002). Recent advances in human sperm pathology. Contraception 65 (4), 283–287. 10.1016/s0010-7824(02)00290-1 12020779

[B5] Baccetti B. Collodel G. Piomboni P. (1996). Apoptosis in human ejaculated sperm cells (notulae seminologicae 9). J. Submicrosc. Cytol. Pathol. 28 (4), 587–596. 8933742

[B6] Bartoov B. Eltes F. Reichart M. Langzam J. Lederman H. Zabludovsky N. (1999). Quantitative ultramorphological analysis of human sperm: fifteen years of experience in the diagnosis and management of male factor infertility. Arch. Androl. 43 (1), 13–25. 10.1080/014850199262698 10445101

[B7] Beare R. Lowekamp B. Yaniv Z. (2018). Image segmentation, registration and characterization in R with SimpleITK. J. Stat. Softw. 86, 8. 10.18637/jss.v086.i08 30288153 PMC6168008

[B8] Berkovitz A. Eltes F. Soffer Y. Zabludovsky N. Beyth Y. Farhi J. (1999). ART success and *in vivo* sperm cell selection depend on the ultramorphological status of spermatozoa. Andrologia 31 (1), 1–8. 10.1046/j.1439-0272.1999.00229.x 9949882

[B9] Björndahl L. Kirkman Brown J. (2022). The sixth edition of the WHO Laboratory Manual for the Examination and Processing of Human Semen: ensuring quality and standardization in basic examination of human ejaculates. Fertil. Steril. 117 (2), 246–251. 10.1016/j.fertnstert.2021.12.012 34986984

[B10] Boyle C. A. Khoury M. J. Katz D. F. Annest J. L. Kresnow M. J. DeStefano F. (1992). The relation of computer-based measures of sperm morphology and motility to male infertility. Epidemiology 3 (3), 239–246. 10.1097/00001648-199205000-00009 1591323

[B11] Bradski G. (2000). The OpenCV library. Dr. Dobb’s J. Softw. Tools. 10.4236/oalib.1108286

[B12] Calvo L. Dennison-Lagos L. Banks S. M. Fugger E. F. Sherins R. J. (1993). Chemical composition and protein source in the capacitation medium significantly affect the ability of human spermatozoa to undergo follicular fluid induced acrosome reaction. Hum. Reprod. 8 (4), 575–580. 10.1093/oxfordjournals.humrep.a138099 8501189

[B13] Cardullo R. A. Baltz J. M. (1991). Metabolic regulation in mammalian sperm: mitochondrial volume determines sperm length and flagellar beat frequency. Cell Motil. Cytoskelet. 19 (3), 180–188. 10.1002/cm.970190306 1878988

[B14] Chandra S. Gourisaria M. K. Gm H. Konar D. Gao X. Wang T. (2022). Prolificacy assessment of spermatozoan via state-of-the-art deep learning frameworks. IEEE Access 10, 13715–13727. 10.1109/access.2022.3146334 35291304 PMC8920051

[B15] Chazotte B. (2009). Labeling mitochondria with fluorescent dyes for imaging. Cold Spring Harb. Protoc. 4 (6), pdb.prot4948. 10.1101/pdb.prot4948 20147179

[B16] Chemes E. H. Rawe Y. V. (2003). Sperm pathology: a step beyond descriptive morphology. Origin, characterization and fertility potential of abnormal sperm phenotypes in infertile men. Hum. Reprod. Update 9 (5), 405–428. 10.1093/humupd/dmg034 14640375

[B17] Chemes H. E. Alvarez S. C. (2012). Tales of the tail and sperm head aches: changing concepts on the prognostic significance of sperm pathologies affecting the head, neck and tail. Asian J. Androl. 14 (1), 14–23. 10.1038/aja.2011.168 22198630 PMC3735144

[B18] Chemes H. E. Olmedo S. B. Carrere C. Oses R. Carizza C. Leisner M. (1999b). Ultrastructural pathology of the sperm flagellum: association between flagellar pathology and fertility prognosis in severely asthenozoospermic men. J. Urol. 161 (5), 1725. 10.1016/s0022-5347(05)69031-3 9806277

[B19] Chemes H. E. Puigdomenech E. T. Carizza C. Olmedo S. B. Zanchetti F. Hermes R. (1999a). Acephalic spermatozoa and abnormal development of the head-neck attachment: a human syndrome of genetic origin. Hum. Reprod. 14 (7), 1811–1818. 10.1093/humrep/14.7.1811 10402395

[B20] Coetzee K. Kruge T. F. Lombard C. J. (1998). Predictive value of normal sperm morphology: a structured literature review. Hum. Reprod. Update 4 (1), 73–82. 10.1093/humupd/4.1.73 9622414

[B21] Collodel G. Federico M. G. Pascarelli N. A. Geminiani M. Renieri T. Moretti E. (2011). A case of severe asthenozoospermia: a novel sperm tail defect of possible genetic origin identified by electron microscopy and immunocytochemistry. Fertil. Steril. 95 (1), 289.e11–e16. 10.1016/j.fertnstert.2010.05.029 20579639

[B22] Cummins J. M. Woodall P. F. (1985). On mammalian sperm dimensions. J. Reprod. Fertil. 75 (1), 153–175. 10.1530/jrf.0.0750153 4032369

[B23] Curti G. Skowronek F. Vernochi R. Rodriguez-Buzzi A. L. Rodriguez-Buzzi J. C. Casanova G. (2014). Morphological evaluation of sperm from infertile men selected by magnetic activated cell sorting (MACS). Reprod. Biol. 14 (4), 289–292. 10.1016/j.repbio.2014.07.002 25454495

[B24] Danis R. B. Samplaski M. K. (2019). Sperm morphology: history, challenges, and impact on natural and assisted fertility. Curr. Urol. Rep. 20 (8), 43. 10.1007/s11934-019-0911-7 31203470

[B25] Engel K. M. Springsguth C. H. Grunewald S. (2018). What happens to the unsuccessful spermatozoa? Andrology 6 (2), 335–344. 10.1111/andr.12467 29438593

[B26] Esbert M. Godo A. Soares S. R. Florensa M. Amorós D. Ballesteros A. (2017). Spermatozoa with numerical chromosomal abnormalities are more prone to be retained by Annexin V-MACS columns. Andrology 5 (4), 807–813. 10.1111/andr.12376 28614636

[B27] Escalier D. (2006). Arrest of flagellum morphogenesis with fibrous sheath immaturity of human spermatozoa. Andrologia 38 (2), 54–60. 10.1111/j.1439-0272.2006.00711.x 16529576

[B28] Escalier D. Serres C. (1985). Aberrant distribution of the peri-axonemal structures in the human spermatozoon: possible role of the axoneme in the spatial organization of the flagellar components. Biol. Cell 53 (3), 239–250. 10.1111/j.1768-322x.1985.tb00372.x 3160418

[B29] Esteves S. C. (2022). Evolution of the World Health Organization semen analysis manual: where are we? Nat. Rev. Urol. 19, 439–446. 10.1038/s41585-022-00593-2 35523961

[B30] Fainberg J. Kashanian J. A. (2019). Recent advances in understanding and managing male infertility. F1000Res 8, F1000 Faculty Rev-670. 10.12688/f1000research.17076.1 PMC6524745 31143441

[B31] Fernández J. L. Muriel L. Goyanes V. Segrelles E. Gosálvez J. Enciso M. (2005). Simple determination of human sperm DNA fragmentation with an improved sperm chromatin dispersion test. Fertil. Steril. 84 (4), 833–842. 10.1016/j.fertnstert.2004.11.089 16213830

[B32] Folgerø T. Bertheussen K. Lindal S. Torbergsen T. Øian P. (1993). Andrology: mitochondrial disease and reduced sperm motility. Hum. Reprod. 8 (11), 1863–1868. 10.1093/oxfordjournals.humrep.a137950 8288752

[B33] Gage M. J. (1998). Mammalian sperm morphometry. Proc. Biol. Sci. 265 (1391), 97–103. 10.1098/rspb.1998.0269 9474794 PMC1688860

[B34] García-Vázquez F. A. Gadea J. Matás C. Holt W. V. (2016). Importance of sperm morphology during sperm transport and fertilization in mammals. Asian J. Androl. 18 (6), 844–850. 10.4103/1008-682X.186880 27624988 PMC5109874

[B35] Grow D. R. Oehninger S. Seltman H. J. Toner J. P. Swanson R. J. Kruger T. F. (1994). Sperm morphology as diagnosed by strict criteria: probing the impact of teratozoospermia on fertilization rate and pregnancy outcome in a large *in vitro* fertilization population. Fertil. Steril. 62 (3), 559–567. 10.1016/s0015-0282(16)56946-5 8062953

[B36] Grunewald S. Fitzl G. Springsguth C. (2017). Induction of ultra-morphological features of apoptosis in mature and immature sperm. Asian J. Androl. 19 (5), 533–537. 10.4103/1008-682X.180974 27270340 PMC5566845

[B37] Grunewald S. Paasch U. (2013). Sperm selection for ICSI using annexin V. Methods Mol. Biol. 927, 257–262. 10.1007/978-1-62703-038-0_23 22992920

[B38] Ho H. C. Wey S. (2007). Three dimensional rendering of the mitochondrial sheath morphogenesis during mouse spermiogenesis. Microsc. Res. Tech. 70 (8), 719–723. 10.1002/jemt.20457 17457821

[B39] Irigoyen P. Pintos-Polasky P. Rosa-Villagran L. Skowronek M. F. Cassina A. Sapiro R. (2022). Mitochondrial metabolism determines the functional status of human sperm and correlates with semen parameters. Front. Cell Dev. Biol. 10, 926684. 10.3389/fcell.2022.926684 36111336 PMC9468643

[B40] Kapur J. N. Sahoo P. K. Wong A. K. C. (1985). A new method for gray-level picture thresholding using the entropy of the histogram. Comput. Vis. Graph. Image Process. 29 (3), 140–185. 10.1016/s0734-189x(85)90156-2

[B41] Karbowski M. Kurono C. Wozniak M. Ostrowski M. Teranishi M. Nishizawa Y. (1999). Free radical-induced megamitochondria formation and apoptosis. Free Radic. Biol. Med. 26 (3-4), 396–409. 10.1016/s0891-5849(98)00209-3 9895232

[B42] Keegan B. R. Barton S. Sanchez X. Berkeley A. S. Krey L. C. Grifo J. (2007). Isolated teratozoospermia does not affect *in vitro* fertilization outcome and is not an indication for intracytoplasmic sperm injection. Fertil. Steril. 88 (6), 1583–1588. 10.1016/j.fertnstert.2007.01.057 17448467

[B43] Khatun A. Rahman M. S. Pang M. G. (2018). Clinical assessment of the male fertility. Obstet. Gynecol. Sci. 61 (2), 179–191. 10.5468/ogs.2018.61.2.179 29564308 PMC5854897

[B44] Kruger T. F. Menkveld R. Stander F. S. Lombard C. J. Van der Merwe J. P. van Zyl J. A. (1986). Sperm morphologic features as a prognostic factor in *in vitro* fertilization. Fertil. Steril. 46 (6), 1118–1123. 10.1016/s0015-0282(16)49891-2 2946611

[B45] Lalonde L. Langlais J. Antaki P. Chapdelaine A. Roberts K. D. Bleau G. (1988). Male infertility associated with round-headed acrosomeless spermatozoa. Obstet. Gynecol. Surv. 43 (9), 561–562. 10.1097/00006254-198809000-00020 3338587

[B46] Lehti M. S. Sironen A. (2017). Formation and function of sperm tail structures in association with sperm motility defects. Biol. Reprod. 97 (4), 522–536. 10.1093/biolre/iox096 29024992

[B47] Lin H. L. Chen Y. H. Chen L. R. (2019). Application of Annexin V magnetic beads enriches boar sperm of high quality. Theriogenology 137, 132. 10.1016/j.theriogenology.2019.05.065

[B48] Mahali M. I. Leu J. S. Darmawan J. T. Avian C. Bachroin N. Prakosa S. W. (2023). A dual architecture fusion and AutoEncoder for automatic morphological classification of human sperm. Sensors (Basel) 23 (14), 6613. 10.3390/s23146613 37514907 PMC10385996

[B49] Marín R. Chang V. (2021). Impact of transfer learning for human sperm segmentation using deep learning. Comput. Biol. Med. 136 (104687), 104687. 10.1016/j.compbiomed.2021.104687 34364259

[B50] Menkveld R. Holleboom C. A. G. Rhemrev J. P. T. (2011). Measurement and significance of sperm morphology. Asian J. Androl. 13 (1), 59–68. 10.1038/aja.2010.67 21076438 PMC3739393

[B51] Menkveld R. Stander F. S. Kotze T. J. Kruger T. F. van Zyl J. A. (1990). The evaluation of morphological characteristics of human spermatozoa according to stricter criteria. Hum. Reprod. 5 (5), 586–592. 10.1093/oxfordjournals.humrep.a137150 2394790

[B52] Morales P. Katz D. F. Overstreet J. W. Samuels S. J. Chang R. J. (1988). The relationship between the motility and morphology of spermatozoa in human semen. J. Androl. 9 (4), 241–247. 10.1002/j.1939-4640.1988.tb01045.x 3182394

[B53] Moretti E. Collodel G. Scapigliati G. Cosci I. Sartini B. Baccetti B. (2005). ‘Round head' sperm defect. Ultrastructural and meiotic segregation study sperm defect. Ultrastruct. meiotic Segreg. study. J Submicrosc Cytol Pathol 37 (3-4), 297–303. 16612973

[B54] Moretti E. Pascarelli N. A. Federico M. G. Renieri T. Collodel G. (2008). Abnormal elongation of midpiece, absence of axoneme and outer dense fibers at principal piece level, supernumerary microtubules: a sperm defect of possible genetic origin? Fertil. Steril. 90 (4), 1201.e3–e8. 10.1016/j.fertnstert.2007.11.050 18291377

[B55] Mossman J. A. Pearson J. T. Moore H. D. Pacey A. A. (2013). Variation in mean human sperm length is linked with semen characteristics. Hum. Reprod. 28 (1), 22–32. 10.1093/humrep/des382 23108349

[B56] Mundy A. J. Ryder T. A. Edmonds D. K. (1995). Asthenozoospermia and the human sperm mid-piece. Hum. Reprod. 10 (1), 116–119. 10.1093/humrep/10.1.116 7745038

[B57] Muratori M. Piomboni P. Baldi E. Filimberti E. Pecchioli P. Moretti E. (2000). Functional and ultrastructural features of DNA-fragmented human sperm. J. Androl. 21 (6), 903–912. 10.1002/j.1939-4640.2000.tb03421.x 11105917

[B58] Olson G. E. Winfrey V. P. (1990). Mitochondria-cytoskeleton interactions in the sperm midpiece. J. Struct. Biol. 103 (1), 13–22. 10.1016/1047-8477(90)90081-m 2397142

[B60] Otani H. Tanaka O. Kasai K. Yoshioka T. (1988). Development of mitochondrial helical sheath in the middle piece of the mouse spermatid tail: regular dispositions and synchronized changes. Anat. Rec. 222 (1), 26–33. 10.1002/ar.1092220106 3189885

[B61] Pelliccione F. Micillo A. Cordeschi G. D’Angeli A. Necozione S. Gandini L. (2011). Altered ultrastructure of mitochondrial membranes is strongly associated with unexplained asthenozoospermia. Fertil. Steril. 95 (2), 641–646. 10.1016/j.fertnstert.2010.07.1086 20840880

[B62] Pelzman D. L. Sandlow J. I. (2024). Sperm morphology: evaluating its clinical relevance in contemporary fertility practice. Reprod. Med. Biol. 23 (1), e12594. 10.1002/rmb2.12594 38915912 PMC11194684

[B63] Poot M. Zhang Y. Z. Krämer J. a. Wells K. S. Jones L. J. Hanzel D. K. (1996). Analysis of mitochondrial morphology and function with novel fixable fluorescent stains. J. Histochem Cytochem 44 (12), 1363–1372. 10.1177/44.12.8985128 8985128

[B64] Rawe V. Y. Galaverna G. D. Acosta A. A. Olmedo S. B. Chemes H. E. (2001). Incidence of tail structure distortions associated with dysplasia of the fibrous sheath in human spermatozoa. Hum. Reprod. 16 (5), 879–886. 10.1093/humrep/16.5.879 11331633

[B65] Rawe V. Y. Galaverna G. D. Olmedo S. B. Acosta A. A. Chemes H. E. (2000). Mitochondrial sheath configuration in abnormal human sperm. Fertil. Steril. 74 (3), S131. 10.1016/s0015-0282(00)01092-x

[B66] Rawe V. Y. Hermes R. Nodar F. N. Fiszbajn G. Chemes H. E. (2007). Results of intracytoplasmic sperm injection in two infertile patients with abnormal organization of sperm mitochondrial sheaths and severe asthenoteratozoospermia. Fertil. Steril. 88 (3), 649–653. 10.1016/j.fertnstert.2006.12.074 17481617

[B67] Reichart M. Eltes F. Soffer Y. Zigenreich E. Yogev L. Bartoov B. (2009). Sperm ultramorphology as a pathophysiological indicator of spermatogenesis in males suffering from varicocele. Andrologia 32 (3), 139–145. 10.1046/j.1439-0272.2000.00355.x 10863968

[B68] Riordon J. McCallum C. Sinton D. (2019). Deep learning for the classification of human sperm. Comput. Biol. Med. 111 (103342), 103342. 10.1016/j.compbiomed.2019.103342 31279166

[B69] Said T. M. Grunewald S. Paasch U. Rasch M. Agarwal A. Glander H. J. (2005). Effects of magnetic-activated cell sorting on sperm motility and cryosurvival rates. Fertil. Steril. 83 (5), 1442–1446. 10.1016/j.fertnstert.2004.11.052 15866582

[B71] Schindelin J. Arganda-Carreras I. Frise E. Kaynig V. Longair M. Pietzsch T. (2012). Fiji: an open-source platform for biological-image analysis. Nat. Methods 9 (7), 676–682. 10.1038/nmeth.2019 22743772 PMC3855844

[B72] Shaker F. Monadjemi S. A. Alirezaie J. (2017). “Classification of human sperm heads using elliptic features and LDA,” in 2017 3rd international conference on pattern recognition and image analysis (IPRIA) (IEEE). 10.1109/pria.2017.7983036

[B73] Shannon C. E. (1948). A mathematical theory of communication. Bell Syst. Tech. J. 27 (3), 379–423. 10.1002/j.1538-7305.1948.tb01338.x

[B74] Skowronek F. Casanova G. Alciaturi J. Capurro A. Cantu L. Montes J. M. (2012). DNA sperm damage correlates with nuclear ultrastructural sperm defects in teratozoospermic men. Andrologia 44 (1), 59–65. 10.1111/j.1439-0272.2010.01106.x 21592172

[B75] Skowronek M. F. Alciaturi J. Casanova G. Capurro A. Montes J. M. Sapiro R. (2010). Value of quantitative ultramorphological sperm analysis in infertile men. Reprod. Biol. 10 (2), 125–139. 10.1016/s1642-431x(12)60055-2 20668504

[B76] Skowronek M. F. Pietroroia S. Silvera D. Ford M. Cassina A. Lecumberry F. (2025). Morphometric analysis of the sperm midpiece during capacitation. Tissue Cell 95 (102866), 102866. 10.1016/j.tice.2025.102866 40157222

[B77] Teranishi M. Spodonik J. H. Karbowski M. Kurono C. Soji T. Wakabayashi T. (2000). Swelling of free-radical-induced megamitochondria causes apoptosis. Exp. Mol. Pathol. 68 (2), 104–123. 10.1006/exmp.1999.2288 10716914

[B78] van der Merwe F. H. Kruger T. F. Oehninger S. C. Lombard C. J. (2005). The use of semen parameters to identify the subfertile male in the general population. Gynecol. Obstet. Invest 59 (2), 86–91. 10.1159/000082368 15572878

[B79] van der Walt S. Schönberger J. L. Nunez-Iglesias J. Boulogne F. Warner J. D. Yager N. (2014). scikit-image: image processing in Python. PeerJ 2, e453. 10.7717/peerj.453 25024921 PMC4081273

[B80] Van Rossum G. Drake F. L. Jr (2009). Python 3 reference manual. Createspace, 244. Available online at https://dl.acm.org/doi/book/10.5555/1593511.

[B81] Van Waart J. Kruger T. F. Lombard C. J. Ombelet W. (2001). Predictive value of normal sperm morphology in intrauterine insemination (IUI): a structured literature review. Hum. Reprod. Update 7 (5), 495–500. 10.1093/humupd/7.5.495 11556497

[B59] World Health Organization (2021). WHO laboratory manual for the examination and processing of human semen,” in Who laboratory manual for the examination and processing of human semen.

[B83] Yoon Y. Wang L. (2015). Morphological control of mitochondrial bioenergetics. Front. Biosci. 20 (2), 229–246. 10.2741/4306 PMC4618469 25553448

[B84] Zhou W. J. Huang C. Jiang S. H. Ji X. R. Gong F. Fan L. Q. (2021). Influence of sperm morphology on pregnancy outcome and offspring in *in vitro* fertilization and intracytoplasmic sperm injection: a matched case-control study. Asian J. Androl. 23 (4), 421–428. 10.4103/aja.aja_91_20 33533739 PMC8269829

