# Supplementary figures and images for: Mitochondrial morphology in fertile and infertile men: image processing and morphometric analysis of the sperm midpiece
Source: Front Cell Dev Biol. 2025 Jun 10;13:1609081. doi: 10.3389/fcell.2025.1609081 (PMC12186058; doi:10.3389/fcell.2025.1609081)

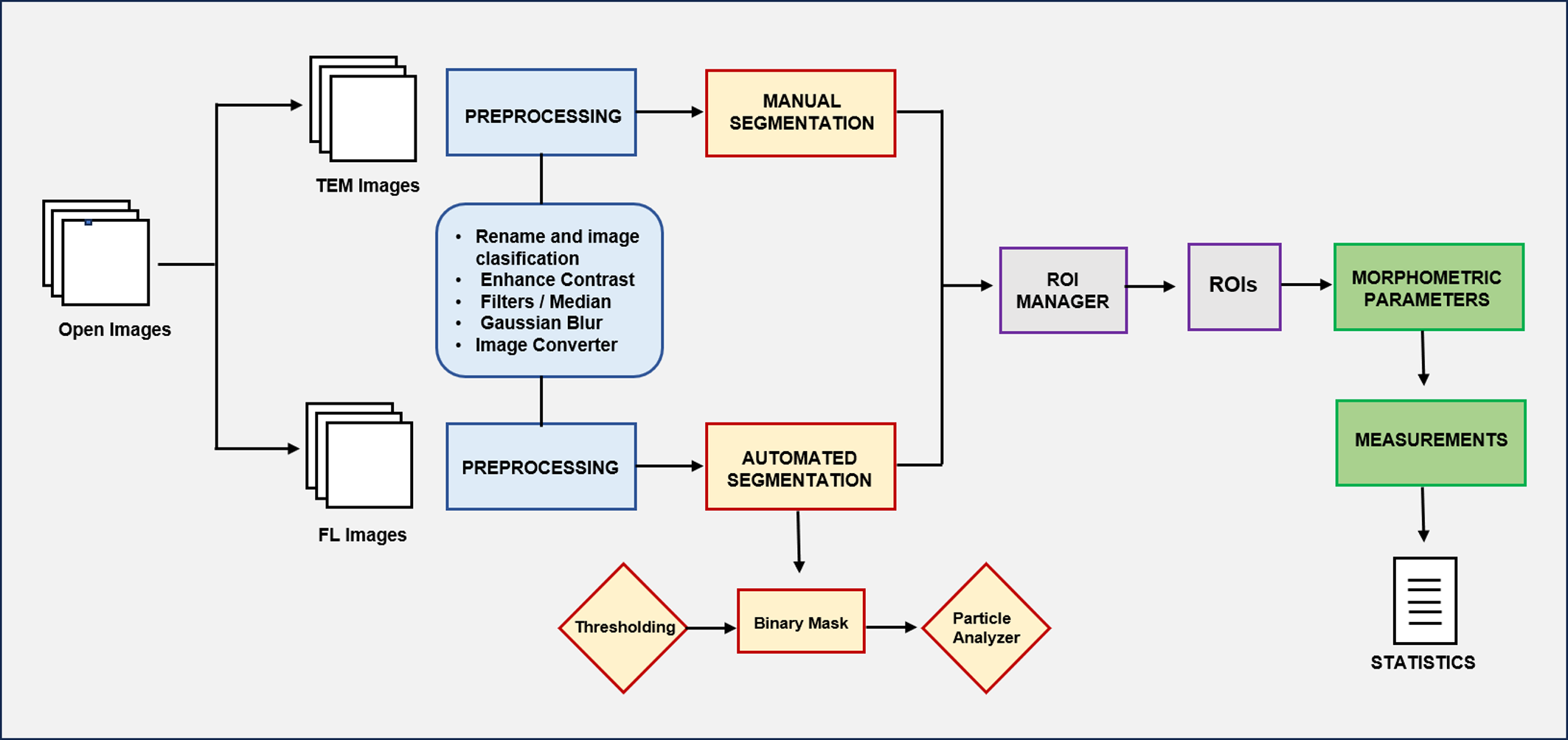

Supplement: Supplementary file 1 [file Image1.tif]
